# Preparation of photothermal responsive, antibacterial hydrogel by using PVA-Alg and silver nanofibers as building blocks

**DOI:** 10.3389/fbioe.2023.1222723

**Published:** 2023-06-20

**Authors:** Jiaxin Ding, Binbin Gao, Xifan Mei

**Affiliations:** Third Affiliated Hospital, Jinzhou Medical University, Jinzhou, China

**Keywords:** Ag nanofibers, biomaterials, nanomedicine, antibacterial hydrogel, photothermal therapy

## Abstract

**Introduction:** Photothermal responsive, antimicrobial hydrogels are very attractive and have great potential in the field of tissue engineering. The defective wound environment and metabolic abnormalities in diabetic skin would lead to bacterial infections. Therefore, multifunctional composites with antimicrobial properties are urgently needed to improve the current therapeutic outcomes of diabetic wounds. We prepared an injectable hydrogel loaded with silver nanofibers for efficient and sustained bactericidal activity.

**Methods:** To construct this hydrogel with good antimicrobial activity, homogeneous silver nanofibers were first prepared by solvothermal method and then dispersed in PVA-lg solution. After homogeneous mixing and gelation, injectable hydrogels (Ag@H) wrapped with silver nanofibers were obtained.

**Results:** By virtue of Ag nanofibers, Ag@H exhibited good photothermal conversion efficiency and good antibacterial activity against drug-resistant bacteria, while the *in vivo* antibacterial also showed excellent performance. The results of antibacterial experiments showed that Ag@H had significant bactericidal effects on MRSA and *E. coli* with 88.4% and 90.3% inhibition rates, respectively.

**Discussion:** The above results indicate that Ag@H with photothermal reactivity and antibacterial activity is very promising for biomedical applications, such as wound healing and tissue engineering.

## 1 Introduction

In recent years, the misuse of antibiotics has led to the emergence of drug-resistant bacteria, and the development of new antimicrobial agents has become a current urgent need (Liu and Rose, 1994; Hussain et al., 2017; Leighow et al., 2020). And nanomaterials have attracted a lot of attention with their unique physical and chemical materials, especially metal nanomaterials. Due to their unique properties, silver nanomaterials have been widely used in optics, electronics, and catalysis (Amjadi et al., 2014; Chen et al., 2016; Satyanarayana et al., 2019), and in addition, they have received a lot of attention in biomedical and antibacterial fields because of their high antibacterial activity (Jatoi et al., 2019; Shukla et al., 2019) and their inhibitory effect on cancer cells (Lin et al., 2021). Silver nanofibers (Ag NWs) are one of the more important silver nanomaterials (Ren et al., 2022), which have a good photothermal conversion efficiency and can be used as photothermal agents for photothermal therapy (Bian et al., 2018; Dong et al., 2022), and under the irradiation of near-infrared light, silver nanofibers can produce a rapid temperature response, and as the temperature increases, the permeability of the cell membrane gradually increases, eventually leading to bacterial death (Cui et al., 2017).

Hydrogels are soft materials consisting of three-dimensional cross-linked networks that act as support scaffolds containing large amounts of water and are very similar to biological tissues such as skin, muscle, and cartilage, and have been widely used in biosensors (Yao et al., 2022), drug delivery systems (Chen N. et al., 2019), tissue engineering (Ma et al., 2020), and wound dressings (Zhu et al., 2022). Polyvinyl alcohol (PVA) is a water-soluble nonionic polyhydroxy polymer with good biocompatibility and biodegradability. As a polymer gel, it has been widely used in a variety of fields, and hydrogels made of PVA have high tensile and excellent self-healing ability (Cao et al., 2022). Sodium alginate is a linear copolymer with unique biocompatibility, biodegradability and non-toxicity (Martínez-Gómez et al., 2017; Zhang et al., 2022). Combining sodium alginate and PVA results in a hydrogel system with good biocompatibility (Luo et al., 2022).

Here, we prepared an antibacterial hydrogel (Ag@H) by mixing Ag nanofibers with PVA-Alg hydrogel. First, we prepared well-dispersed Ag nanofibers by the solvothermal method, and added them to the hydrogel and mixed them well to obtain the antibacterial hydrogel system. In this work, Ag@H will inherit the antibacterial properties of Ag nanofibers with good photothermal conversion efficiency, reaching about 47°C to kill bacteria under the irradiation of near-infrared light. In addition, the improvement of the hydrogel properties by the incorporation of Ag nanofibers was also investigated. Compared with single silver nanofibers, the presence of silver nanofibers in Ag@H composite hydrogel endows the hydrogel with good photothermal and antimicrobial activities and improves the properties of the hydrogel with better mechanical properties, while the hydrogel can continuously release silver, which makes the composite hydrogel have continuous and efficient antimicrobial activity.

## 2 Experimental section

### 2.1 Materials

The reagents used in this experiment were all of analytical grade (AR) and could be used without purification. Sodium chloride (NaCl), polyvinylpyrrolidone (PVP), silver nitrate (AgNO_3_), ethylene glycol, anhydrous ethanol, polyvinyl alcohol (PVA) and sodium alginate (Alg) were purchased from Shanghai Aladdin Biochemical Technology Co. Ultra-pure water was used throughout the experiments.

### 2.2 Preparation of silver nanofibers

Silver nanofibers were prepared using a modified process from other studies (Zhang et al., 2017), in brief, 0.01 g of sodium chloride solid and 0.0793 g of PVP were added to 10 mL of ethylene glycol solution, followed by the rapid addition of 10 mL of ethylene glycol solution of silver nitrate (0.05 M). The mixed solution was stirred thoroughly, transferred to an autoclave, reacted at 160°C for 4 h, cooled to room temperature, and the precipitate was collected by centrifugation at 6,000 r/min for 10 min and washed three times each with anhydrous ethanol and ultrapure water.

### 2.3 Preparation of Ag@H hydrogel

A certain amount of polyvinyl alcohol was put into 10 mL of ultrapure water to configure a solution with a concentration of 8%, then 0.1 g of sodium alginate powder was added to it, and the mixture was placed in a water bath at 80°C with magnetic stirring for 1 h to dissolve it fully, and then the prepared silver nanofibers were added to obtain Ag@H.

### 2.4 Characterization of materials

The morphology of silver nanofibers was characterized by scanning electron microscopy (SEM, Hitachi, S4800, Tokyo, Japan), the crystal structure of silver nanofibers was tested by X-ray diffraction (XRD, Shimadzu, Kyoto, Japan), and the crystal structure of silver nanofibers was tested using Fourier transform infrared spectroscopy (FTIR. Shimadzu, Kyoto, Japan) was used to obtain infrared spectra from 400 to 4,000 cm^−1^. The cross-sectional morphology of the lyophilized samples was observed using an optical microscope. The compression properties of the hydrogels were evaluated by compressing them at room temperature.

### 2.5 Photothermal properties and photothermal stability of Ag@H

Different treatment groups were added into 1.5 mL centrifuge tubes and irradiated using 808 nm wavelength NIR light for 15 min to record the temperature changes and thermal images, in addition, photothermal cycling experiments were performed with irradiation/no irradiation time of 10 min and repeated for three cycles to evaluate the photostability of Ag@H. The hydrogel lyophilized samples were also irradiated and their maximum temperatures and thermal images were recorded.

### 2.6 Antibacterial performance test

The antibacterial performance of different samples under NIR irradiation was tested by Oxford cup method, and MRSA and *E. coli* were selected. Firstly, the bacterial suspension (100 μL) was evenly dispersed on LB agar medium, and the Oxford cup was placed vertically on the surface of the medium, and 10 μL of different samples were added to the cup. After 10 min of 808 nm laser irradiation, the samples were incubated at 37°C, and the antimicrobial ring diameter was measured after 24 h to evaluate the antimicrobial effect.

### 2.7 Detection of bacterial reactive oxygen species production

The reactive oxygen species detection kit was used to detect MRSA oxidation induced by different samples. MRSA was incubated under different treatment groups for 30 min at 37°C, and then MRSA was incubated with the DCFH-DA probe in the dark for 30 min. Finally, the fluorescent stained images of the probe treatment were observed using a fluorescence microscope.

### 2.8 Biofilm inhibition assay

500 μL of MRSA (5 × 10^8^ CFU/mL) was added to 24-well plates and incubated at 37°C for 48 h to form biofilms, which were rinsed three times with PBS. The groups of materials were then added to the wells and incubated at 37°C with or without NIR laser irradiation. The clear solution was then aspirated and 500 μL of methanol was added to each well for fixation. 20 min later, the methanol was aspirated and dried naturally. Then 200 μL of crystalline violet dye (CV) was added to stain the biofilm, and after 15 min, the unbound crystalline violet was removed by rinsing twice with ultrapure water. Then, 200 μL of anhydrous ethanol solution was added to each well, incubated for 30 min, and the absorbance of each sample at 590 nm was measured by an enzyme marker.

## 3 Results and discussion

### 3.1 Design and properties of Ag@H

**FIGURE 1:**
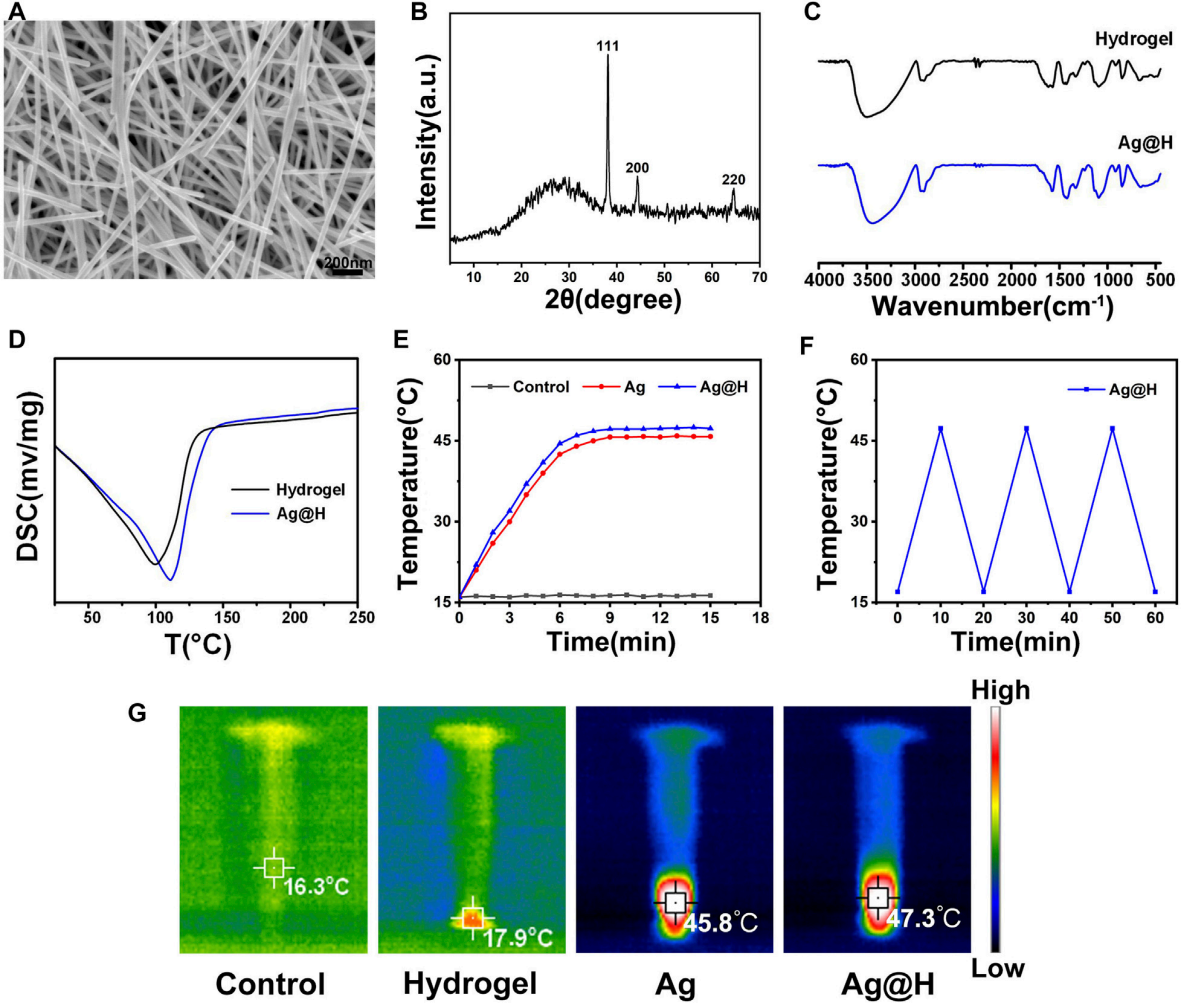
**(A)** SEM image of Ag nanofibers. **(B)** XRD. **(C)** FTIR spectra. **(D)** DSC curves of hydrogel and Ag@H. **(E)** Temperature change curves during laser irradiation. **(F)** Photothermal conversion stability of Ag@H. **(G)** Infrared thermography of control, hydrogel, Ag, and Ag@H.

The prepared Ag nanofibers were characterized by SEM and XRD. As shown in Figure 1A, the smooth surface Ag nanofibers with an average diameter of about 50 nm can be observed by SEM; the diffraction peaks at 38.12°, 44.32°, and 64.54° in Figure 1B correspond to the (111), (200), and (220) crystal surfaces of Ag, respectively, indicating the successful preparation of Ag nanofibers. The FTIR spectra are shown in Figure 1C, where the broad absorption band at 3,440 cm^−1^ is the absorption peak of -OH and 1,560 cm^−1^ represents the stretching vibration of C=O. The uniform dispersion of Ag nanofibers into the hydrogel shows the characteristic absorption peak of the hydrogel intact, indicating that the Ag nanofibers bind well to the hydrogel and do not change its structure. The synthesis and release of Ag nanofibers were also confirmed by UV-vis (Supplementary Figure S1), the absorption peak at 398 nm was attributed to the plasmon resonance of silver nanofibers, and the absorption intensity increased gradually with time, indicating the release of silver. In addition, the differential thermal analysis of the pure hydrogel and the hydrogel loaded with Ag nanofibers (Figure 1D) showed that the maximum heat absorption temperature of Ag@H (110°C) was higher than that of the pure hydrogel (99°C), which might be due to the doping of Ag nanofibers to produce more hydrogen bonds inside the hydrogel and more stable structure of the hydrogel, and also indicated that the hydrogel loaded with Ag nanofibers had better moisturizing effect. To investigate the photothermal properties of Ag@H, different sample solutions were irradiated for 10 min using a near-infrared laser, and temperature changes and thermographic images were recorded (Figures 1E, G), where both Ag and Ag@H exhibited good photothermal properties with temperatures increasing to 45.8°C and 47.3°C, respectively. Subsequently, we investigated the stability of Ag@H under NIR laser, as shown in Figure 1F. After three cycles, the maximum temperature of Ag@H was maintained at about 47°C, indicating that the prepared Ag@H has good photothermal effect and photothermal stability (Chen Z. et al., 2019).

### 3.2 Synthesis and characterization of hydrogels

**FIGURE 2:**
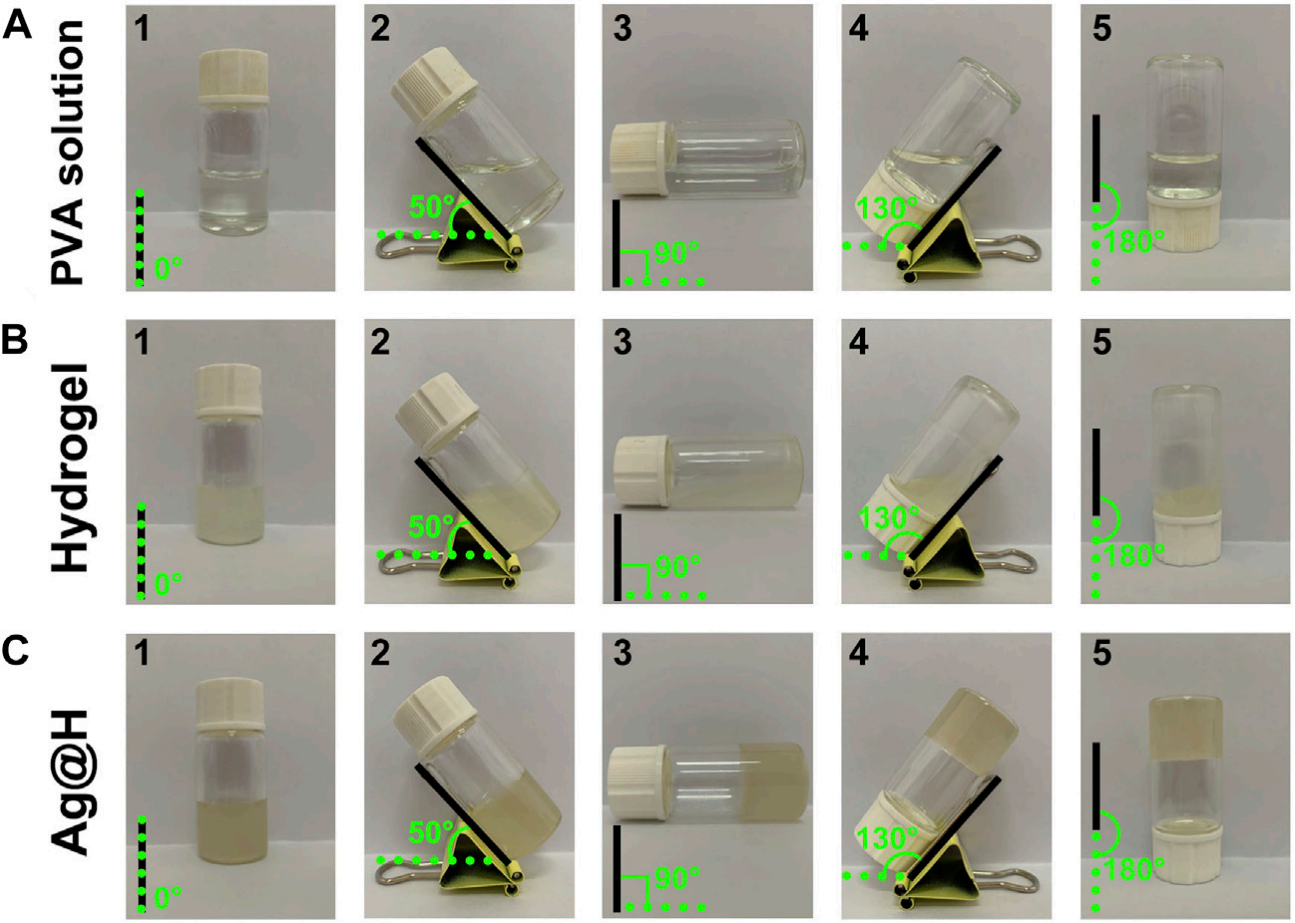
The formation process of hydrogel. **(A)** Aqueous PVA solution. **(B)** Hydrogel. **(C)** Ag@H.

**FIGURE 3:**
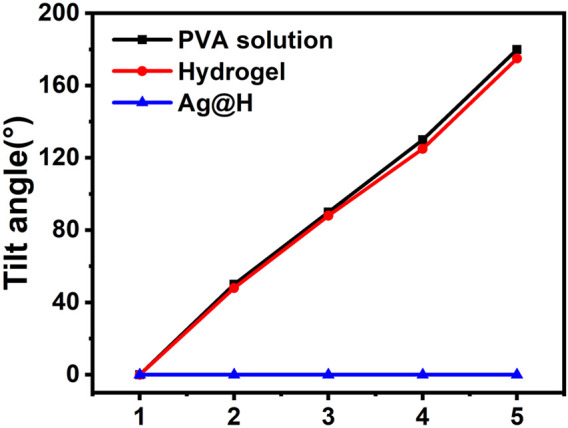
Tilt angle of PVA solution, hydrogel, and Ag@H.

Figure 2 represents the preparation of hydrogel, firstly, take appropriate amount of PVA dissolved in 10 mL of distilled water and dissolve it fully at 90°C, then pour 0.3 g of Alg powder into it, stir it at 70°C and mix it well to get hydrogel solution, then add the prepared Ag nanofibers to get Ag@H. As shown in Figure 2A, before adding Alg, the PVA solution was fluid and could not gel. After Alg was mixed well, the PVA solution gelled, but did not lose its fluidic ability (Figure 2B). With the addition of Ag nanofibers, the hydrogel turned yellow with the interconnection of hydrogen bonds with the PVA surface, and the degree of cross-linking increased, further gelation and thus loss of fluid capacity (Figure 2C), indicating the successful preparation of Ag@H composite hydrogels. The analysis of the tilt angle of the hydrogel also confirms the synthesis of Ag@H (Figure 3).

**FIGURE 4:**
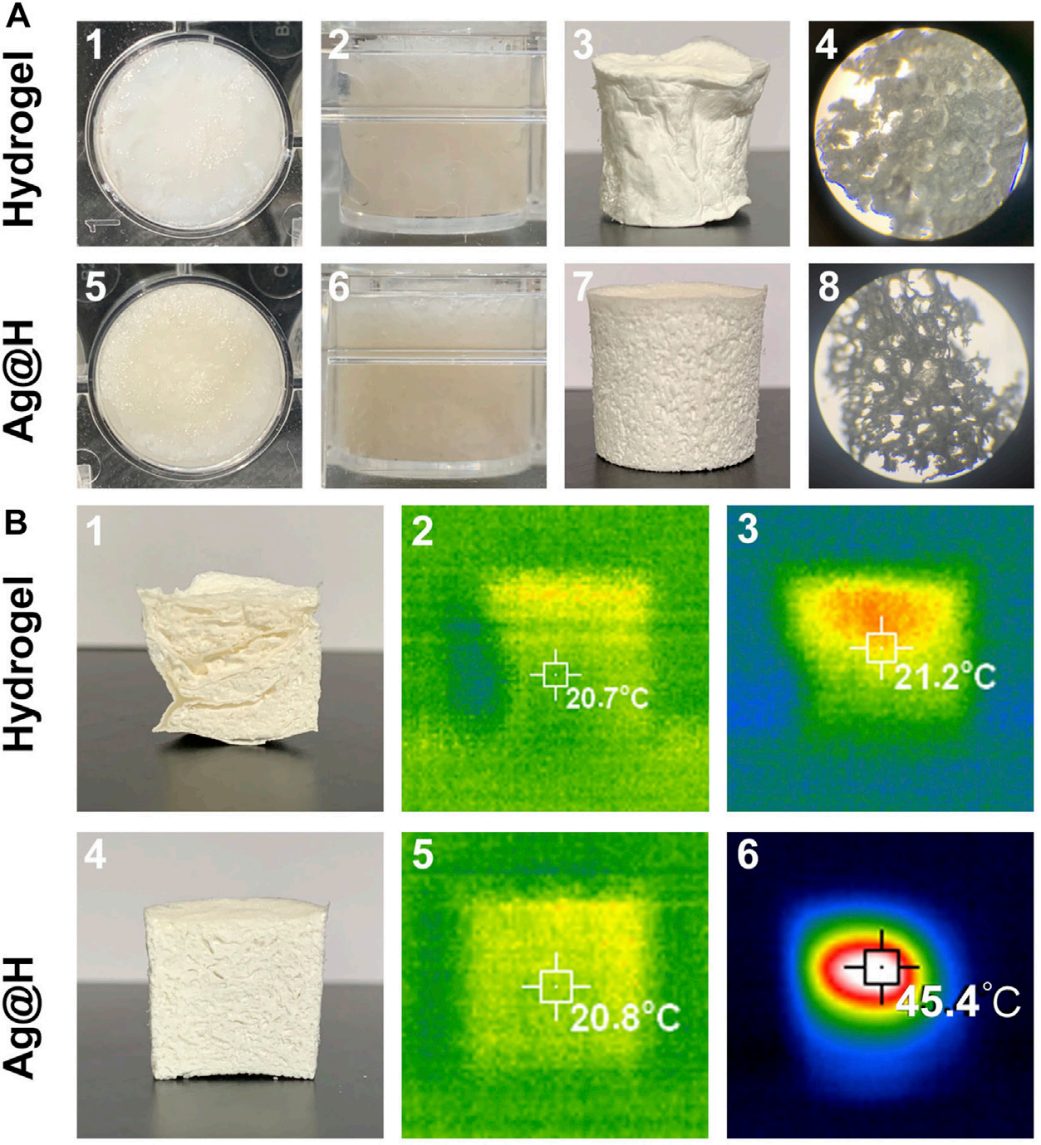
**(A)** Macrostructure of the hydrogel with Ag@H. **(B)** Thermal imaging of hydrogel and Ag@H freeze-dried samples.

When the hydrogels were placed in 24-well plates, a yellowish color of Ag@H was observed relative to the pure hydrogels (Figure 4A 1, 2, 5, 6) due to the doping of Ag nanofibers. After freeze-drying, Ag@H could form regular gel blocks and obvious porous structures could be observed under the microscope (Figure 4A 7, 8), while the pure hydrogels did not have similar porous structures (Figure 4A 3, 4), a phenomenon indicating that the Ag nanofibers had successfully bonded to the hydrogels with significant porosity, and the formation of these pores was due to the sublimation of ice crystals in the primary stage of freeze-drying of the hydrogels. After the freeze-dried hydrogel was irradiated by near infrared laser for 10 min, the highest temperature of Ag@H could reach 45.4°C (Figure 4B), indicating that it still had a good photothermal conversion efficiency.

**FIGURE 5:**
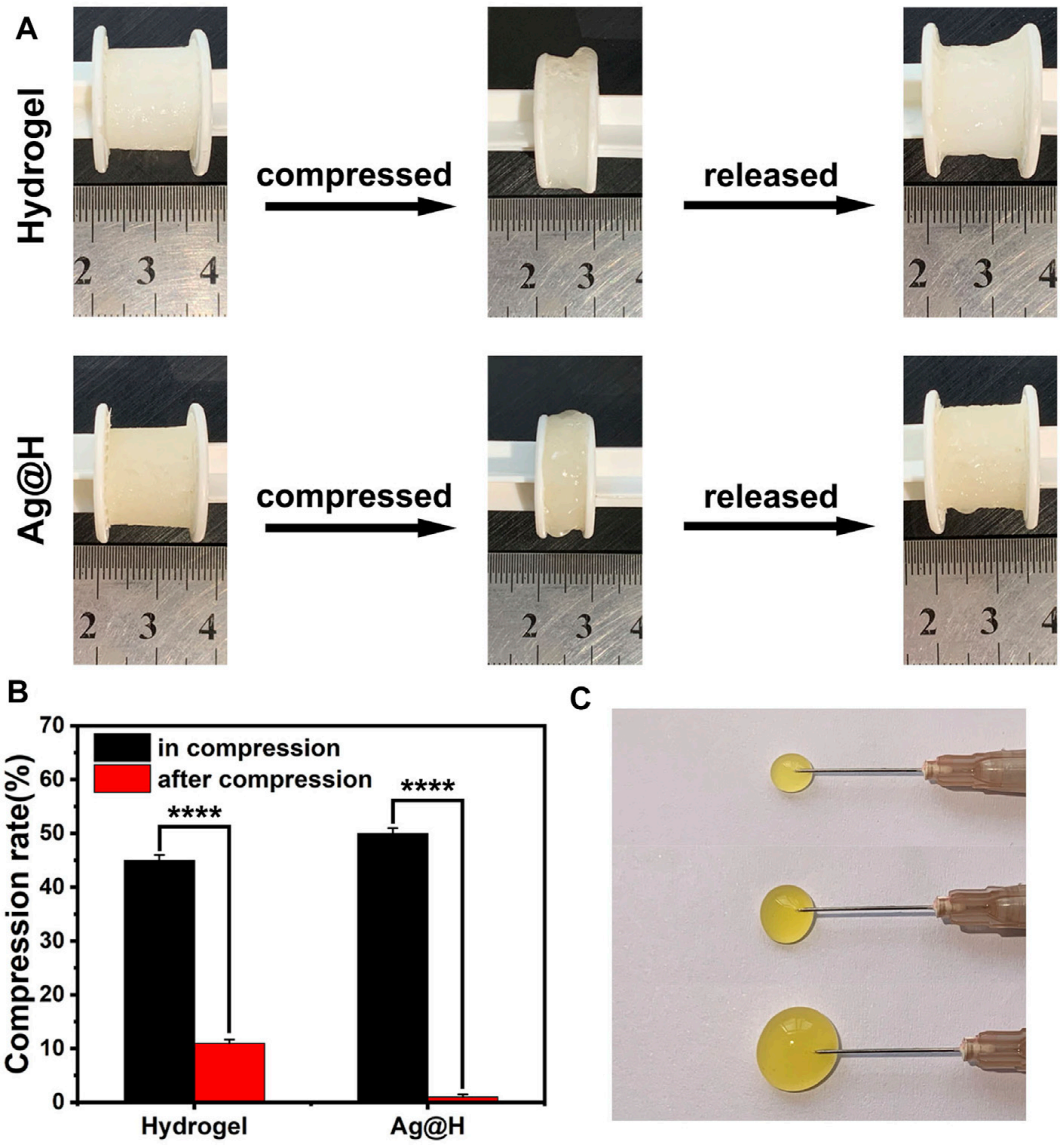
**(A)** Recovery of hydrogel and Ag@H after compression. **(B)** Compressibility of the hydrogel. **(C)** Injectability of the hydrogel.

As shown in Figures 5A, B, the hydrogels have good compressibility, and the compression rates of the hydrogels and Ag@H are 45% and 50%, respectively, when given the same force, and Ag@H can quickly recover its shape after removing the pressure, indicating that the addition of Ag nanofibers can increase the degree of cross-linking to improve the mechanical properties of the hydrogels. Subsequently, the injectability of the hydrogel was investigated, and the hydrogel could be smoothly extruded from the syringe needle (0.45 × 16 mm) without any obstruction (Figure 5C), indicating that the hydrogel has good injectability.

### 3.3 Antimicrobial performance evaluation

**FIGURE 6:**
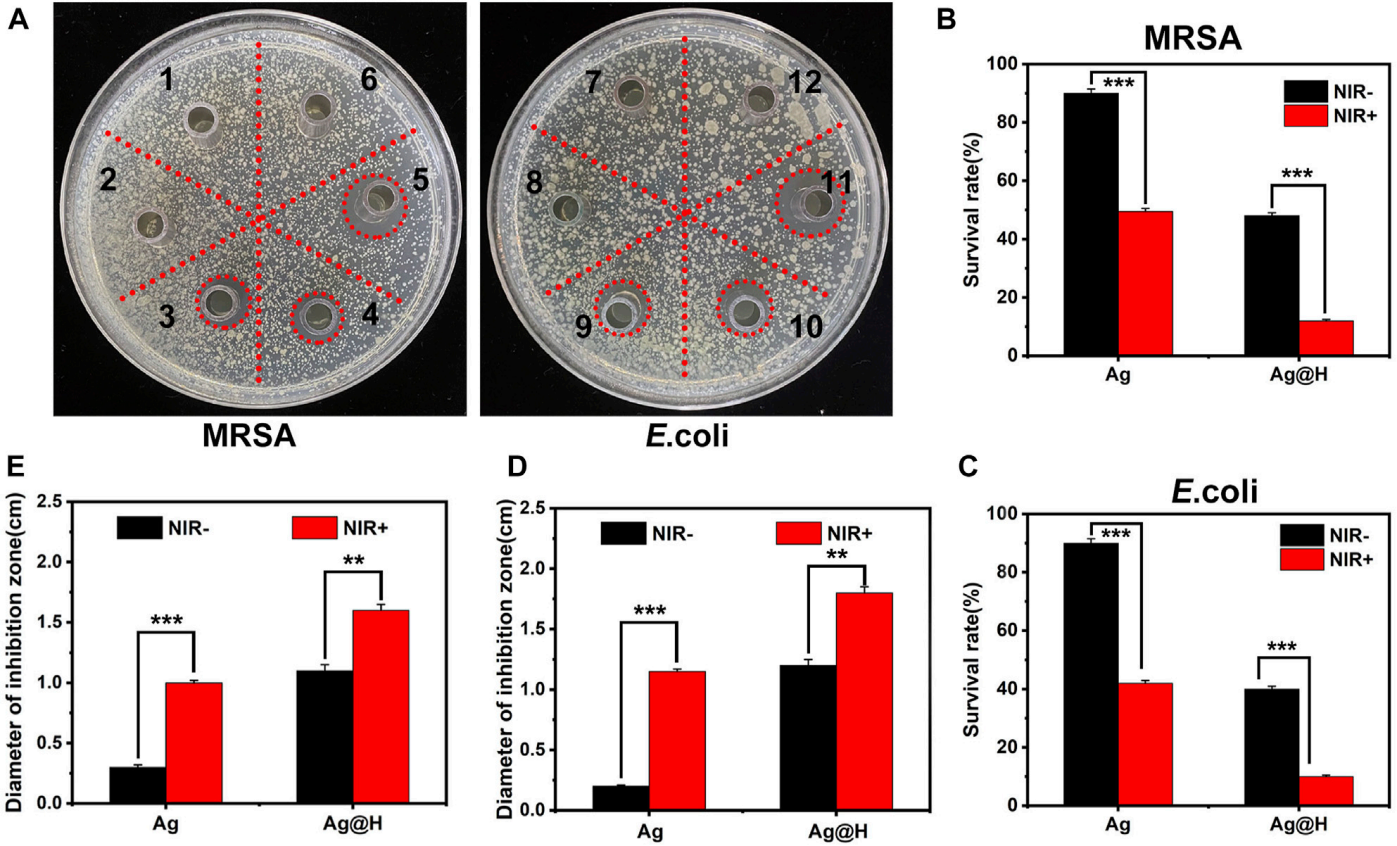
**(A)** Inhibition zones of Ag nanofibers and Ag@H against MRSA and *E*. *coli* under NIR illumination (1, 2, 3 and 7, 8, 9 for Control group, Ag nanofibers group and Ag@H, respectively, and 4, 5, 6 and 10, 11, 12 for Ag@H + NIR, Ag + NIR and Control + NIR, respectively). **(B, C)** Survival rates of MRSA and *E*. *coli*. **(D, E)** Quantitative analysis results of the diameter of the inhibition zone of MRSA and *E*. *coli*.

Firstly, the antibacterial performance of different groups was evaluated by Oxford cup method (Figure 6A), and after 8 h incubation under laser irradiation in each group, it could be observed that no inhibition zone was seen in each blank group and Ag-treated group, while obvious inhibition zones could be observed in Ag + NIR and Ag@H treated groups, among which the largest inhibition zone area was observed in Ag@H + NIR group, and the diameters of corresponding MRSA and *E*. *coli* were 1.6 and 1.8 cm, respectively (Figures 6D, E). The above results showed that the control group and Ag nanofibers without laser irradiation had no inhibitory effect on bacteria, while Ag nanofibers with laser irradiation showed significant inhibitory effect on MRSA and *E*. *coli* with both Ag@H treated groups. The survival rates of bacteria in Ag and Ag@H treatment groups were subsequently calculated (Figures 6B, C), and the survival rates of MRSA and *E*. *coli* under Ag@H + NIR was only 12% and 10%. The above results indicated that Ag@H had a good inhibitory effect on bacteria under NIR light irradiation.

### 3.4 Antibacterial mechanism

**FIGURE 7:**
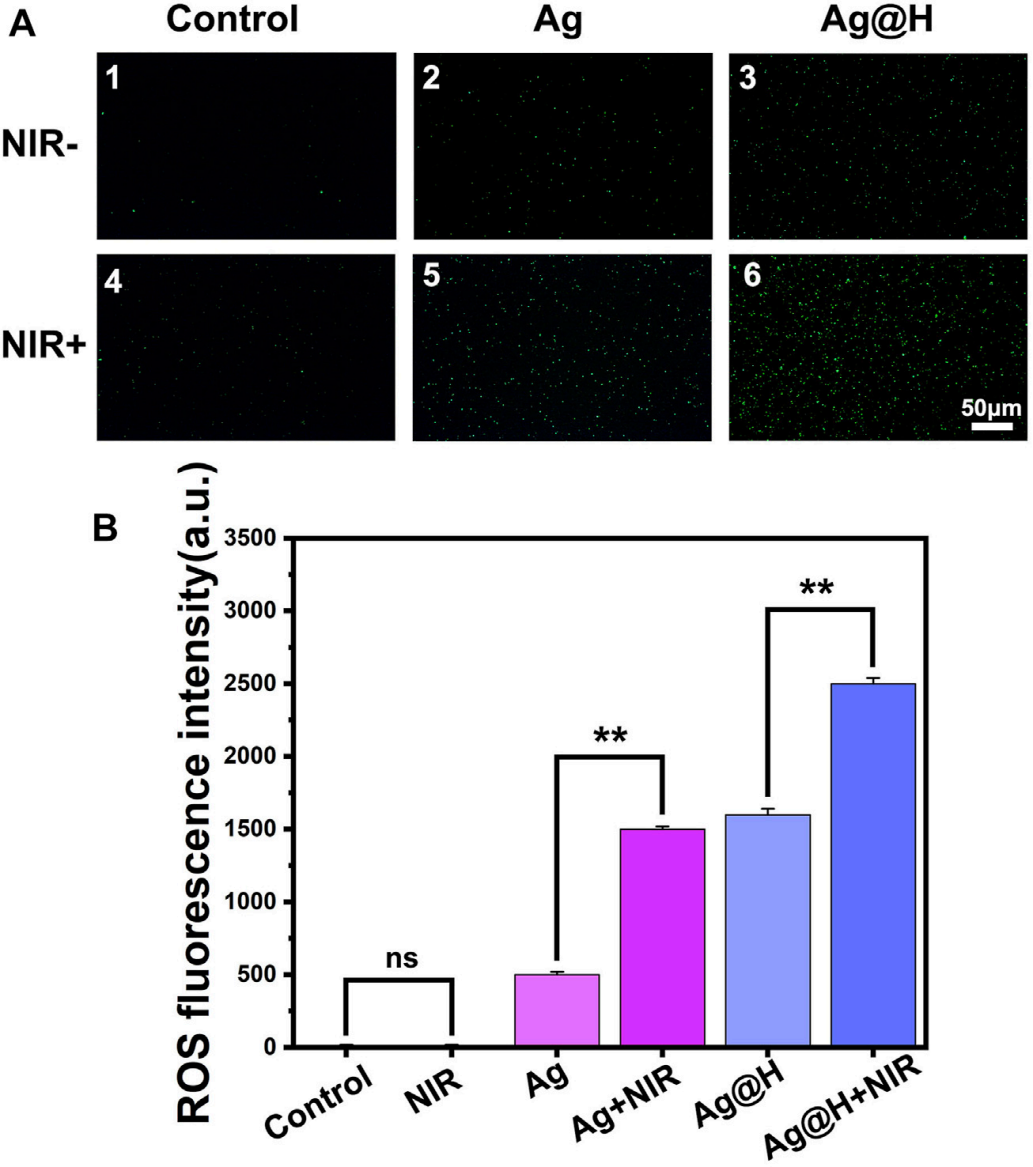
**(A)** ROS fluorescence detection images by DCFH-DA processing. **(B)** Quantitative analysis of ROS fluorescence.

Based on the above results, the production of ROS in bacteria under different treatment groups was monitored using the DCFH-DA fluorescent probe, as shown in Figure 7A. Significant green fluorescence was observed in both blank groups, while a large amount of green fluorescence could be observed in the Ag@H group under NIR light irradiation, which was due to the high temperature generated by PTT leading to an oxidative stress environment that would promote ROS production, and quantitative fluorescence analysis also confirmed the high level of ROS (Figure 7B).

**FIGURE 8:**
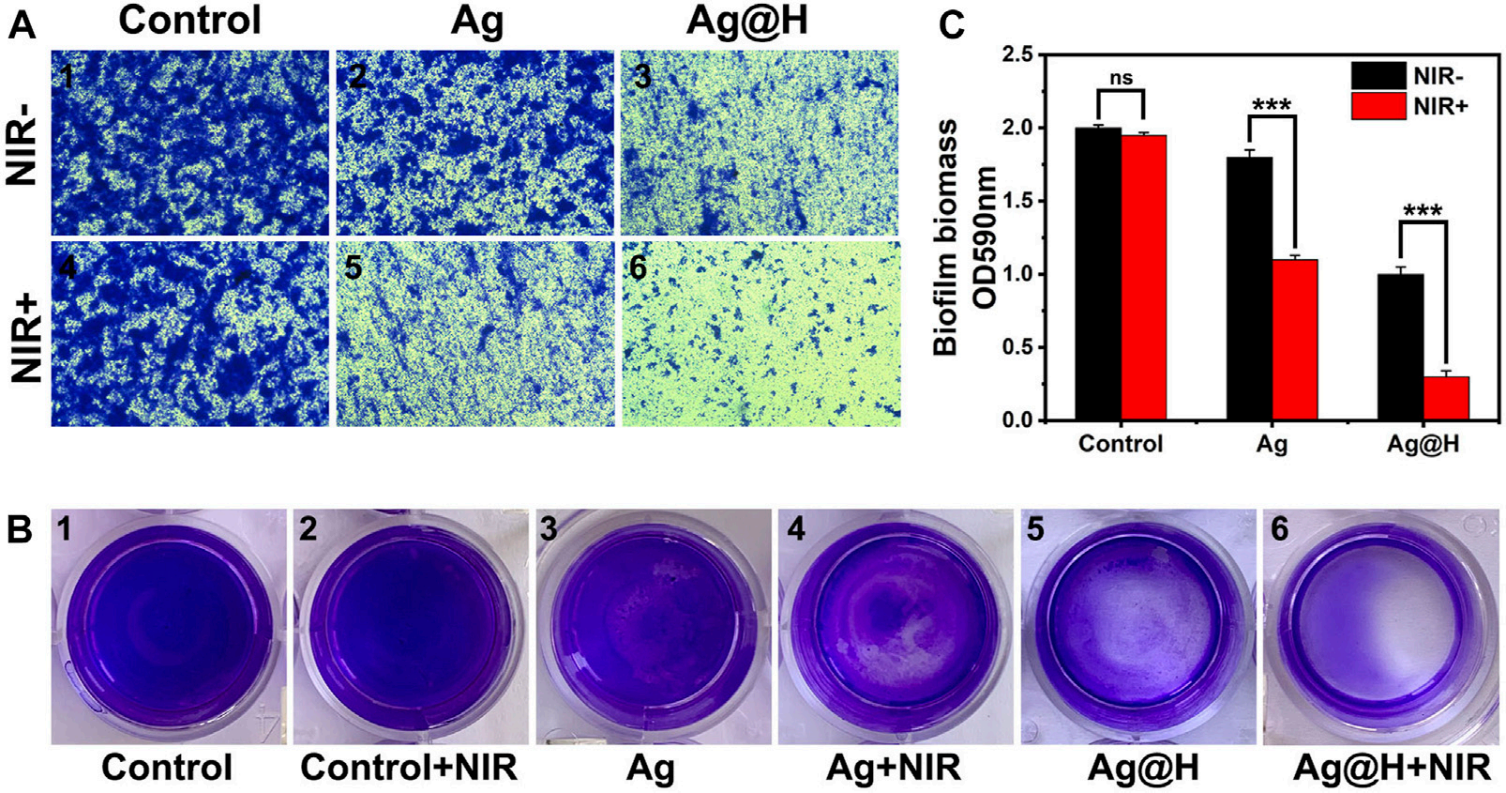
**(A)** Microscopic observation of MRSA biofilm stained with crystalline violet. **(B)** Images of MRSA biofilm stained with crystalline violet. **(C)** Quantitative analysis of bacterial biofilm.

Biofilm formation can hinder antibacterial effectiveness, and bacteria in biofilms are more difficult to remove than free bacteria. The crystalline violet dye can bind to the molecules on the surface of the bacterial biofilm (Huang et al., 2021). As can be observed from Figures 8A, B, the control group and the Ag nanowire group without NIR light irradiation had the darkest blue color and formed a more complete biofilm, while the Ag@H + NIR group was the lightest blue color. Meanwhile, the absorbance of Ag@H + NIR was found to be significantly lower than the other groups by quantitative analysis (Figure 8C), which indicates that Ag@H can destroy the formation of biofilm and has a better bactericidal effect.

## 4 Conclusion

In summary, we prepared an antibacterial hydrogel loaded with Ag nanofibers. Firstly, silver nanofibers with uniform shape, smooth surface and average diameter of about 50 nm were prepared by the solvent thermal method using ethylene glycol as the solvent, and then they were uniformly dispersed into the PVA-Alg hydrogel to synthesize a hydrogel with antibacterial activity (Ag@H). Under near-infrared light, Ag@H can increase the temperature to 47.3°C and inhibit MRSA and *E*. *coli* by 88.4% and 90.3%, respectively. The results showed that the prepared composite hydrogel can effectively kill bacteria and has excellent antibacterial properties.

## Data Availability

The original contributions presented in the study are included in the article/Supplementary Material, further inquiries can be directed to the corresponding author.
