# Supplementary figures and images for: Preparation of photothermal responsive, antibacterial hydrogel by using PVA-Alg and silver nanofibers as building blocks
Source: Front Bioeng Biotechnol. 2023 Jun 20;11:1222723. doi: 10.3389/fbioe.2023.1222723 (PMC10319420; doi:10.3389/fbioe.2023.1222723)

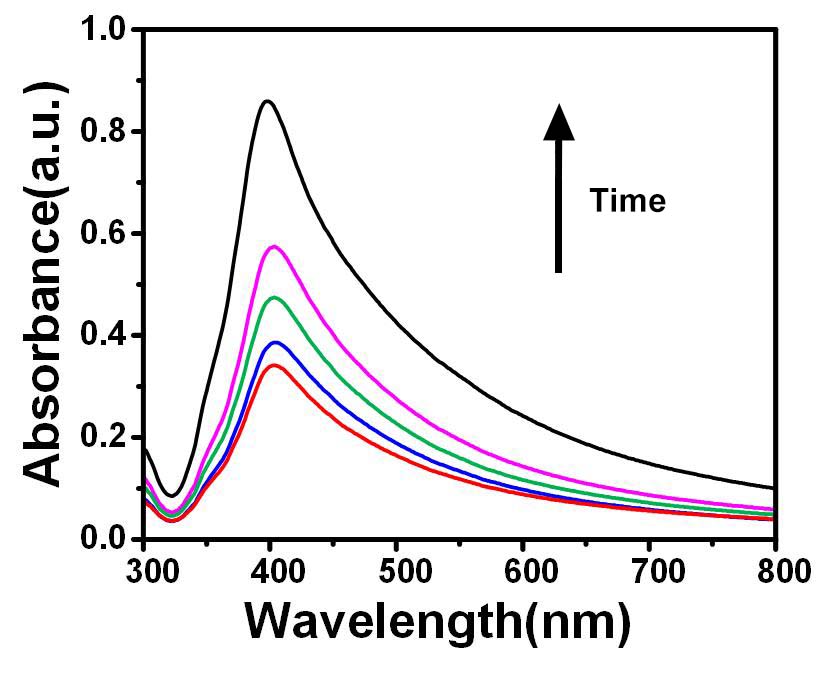


**Figure S1.** Ag release test of Ag@H.

Supplement: Supplementary file 1 [file DataSheet1.docx]
